# Hardness Analysis and Empirical Studies of the Relations among Robustness, Topology and Flow in Dynamic Networks

**DOI:** 10.1371/journal.pone.0145421

**Published:** 2015-12-22

**Authors:** Xing Zhou, Wei Peng, Zhen Xu, Bo Yang

**Affiliations:** National Laboratory for Parallel and Distributed Processing, National University of Defense Technology, Changsha, Hunan, China; Nankai University, CHINA

## Abstract

Network robustness is the ability of a network to maintain performance after disruption, thus it is an important index for network designers to refer to. Every actual network has its own topology structure, flow magnitude (scale) and flow distribution. How the robustness relates to these factors still remains unresolved. To analyze the relations, we first established a robustness problem model, studied the hardness of a special case of the model, and generated a lot of representative network instances. We conducted experiments on these instances, deleting 5% to 50% edges on each instance and found that the robustness of a network has an approximate linearity to its structural entropy and flow entropy, when the correlation coefficient between the structure and flow is fixed. We also found that robustness is unlikely to have a relation to the flow scale and edge scale in our model. The empirical studies thus can provide a way of quickly estimating the robustness of real-world networks by using the regression coefficients we obtained during the experiments. We conducted computation on a real-world dataset and got favorable results, which exhibited the effectiveness of the estimation.

## Introduction

Network robustness refers to the resilience of a network when subjected to pressure and disruption [1]. It is the ability of a network to tolerate accidents and damages to nodes or links. Network providers and defenders attempt to maintain a maximum of network’s availability, whereas malicious attackers try to destroy the network as much as possible. Because of the life-and-death importance for both sides, the studies of the robustness of networks have attracted many researchers’ attention.

At the very beginning, the studies have focused primarily on the relationship between the robustness of a complex network and its topology, because elements, i.e. links and nodes, greatly impact the availability of the network. Elements’ alteration can sharply change the robustness of the network. Barabási’s original work [2] showed that a network’s topological characteristics has a significant influence on its robustness. Researchers consequently found that, scale-free complex networks under the Barabási-Albert (BA) model, which features heterogeneous nodal degree distribution, will be resilient against random failures but fragile towards intentional attacks [2–6]. On the other hand, networks under the Erdős-Rényi (ER) model whose nodal degrees are uniformly distributed, are quite robust against intentional attacks [7, 8]. By rewiring links, one can altering the degree distribution, and therefore can change the robustness of networks. The authors of [9] using simulations found that scale-free networks with “onion structure” are very robust against targeted high degree attacks. Later, more researchers confirmed this finding and applied or adapted this verdict to robust network analysis and design [10–12]. Recently, the deeper causality and the dynamics reasons of this finding were revealed clearer [13, 14].

However, real-world robustness is dependent on not only the topology, but also on the dynamic interaction flow on the network. For example, the breakdown of a node with more flow originated from it will be more serious than breakdown on those of less flow. Matisziw is a pilot to investigate this situation. In [1], he and others studied the robustness of complex networks that have nodal interactions (directed flows, in fact). They empirically analyzed the differences of robustness that are caused by different flow distributions in varying time intervals on the American backbone network. Their results showed that robustness varies temporally and that the critical link set varies spatially over different time intervals. They therefore concluded that a network’s robustness is sensitive to nodal interaction changes. However, they did not tell us how the interaction’s distribution on a network’s topology affects the robustness.

Therefore, in this paper, we attempt to investigate several problems for random networks and scale-free networks: How does the heterogeneity of a flow’s distribution affect the robustness? How does a flow’s distribution on the network’s topology affect the robustness? Do flow size, edge size or node size matter to robustness (Do flow volume, edge number or node number matter)?

Our work presumed the following scenario. An attacker can only remove links of a network. As the attacker has finite attacking resources, he can only remove at most *k* links. His goal is to make the network support the fewest interaction flows after the link removal. This problem is a more generic version of the critical link set problem. The critical link set problem (denoted: CLP) specifies a unit of interaction flow on every pair of node. In [15], CLP has been shown to be NP-complete, so there is unlikely polynomial time complexity exact algorithm that can solve CLP. Therefore, the more generalized robustness problem is also NP-complete. There are many heuristic algorithms for similar problems of CLP. For example, article [16] proposed an exact algorithm for a similar problem: critical clique detection problems; article [17] surveyed the approaches for critical element detection problems. We want to point out that our work differs from those approaches in that our method is an approximate approach and that the results obtained in our paper can be conveniently used to estimating other networks with similar graph size.

Our contributions in this paper include:

- Built up an evaluation model for a network’s robustness with non-uniform interaction flows on it. The model is easier to understand than [1] and can be applied to real world usage.
- Derived out the inapproximability ratio of the robustness problem when the problem shrinks to the critical link set problem (CLP).
- Generated massive representative instances (data) with different topologies, flows and coupling levels to find the relations among robustness, topology and flow.
- Designed optimal and near-optimal optimization algorithms to calculate the generated cases of the model.
- Discovered robustness’ nearly linear relationship to topology complexity and flow complexity and found that robustness may have nothing to do with flow scale, and edge scale when deleting edges by percentage instead of fixed numbers.
- Using the regression coefficients, we applied them to a real world network and got good estimation. There are other papers to estimate the bounds, such as [18]. However, their work has not considered graphs with weighted edges while our paper mainly to address the estimation of weighted graph in a different method.

## Methods

In order to investigate how much network topology, network nodal interaction flows and the two’s coupling level affect network robustness, we first established a mathematical model concerning the total remaining flow in the residual network after a specific deletion. Then, we generated massive instances with varying structure, flow and coupling parameters. Subsequently, we computed 10 cases of optimal value of each instance. However, we found that the optimum computation was time unfeasible because of its NP-completeness. But fortunately, the genetic algorithms (GA) [19] can produce very near results; therefore we adopted a genetic algorithm (GA) to calculate the approximate results. Finally, we derived some relations by analyzing the results we obtained. We in the end applied the numerical findings to real-world practice.

### Model

Studies concerning robustness have used many metrics, such as giant component size, toughness, algebraic connectivity, and natural connectivity [20] to evaluate a network’s structural robustness. In [15], the authors used a metric called “pairwise connectivity”. Pairwise connectivity is the total number of node pairs which are mutually reachable. Recent studies that considered flow used more precise metrics, such as elasticity of robustness [1]. In this paper, we extend pairwise connectivity (denoted: PC) to residual flow. Then, we newly defined a metric, *Robu*, which is equal to residual flow after deletion divided by original overall flow.

Because network robustness is its ability to preserve performance, we want to know how much the residual flow will be if the removal strategy is the best for the attacker, i.e., how much flow the network can preserve after the cleverest deletion, thus avoiding robustness’s dependency on the deletion strategy’s uncertainty. We can model the problem as follows:

Assume an undirected simple weighted graph *G* = (*V*, *E*, *f*), where *V* is the node set, *E* the edge set and f:V×V→N. Let *n* = |*V*| be the total number of nodes and *e* = |*E*| be the number of edges. We denote the interaction flow from *i* to *j* as *f*_*i*, *j*_ or *f*_*ij*_. The “interaction flow from *i* to *j*”, can be viewed as the quantity of flow that *i* sends to *j*. Assume, at the beginning, the graph is all connected, so we can define the original total supported interaction flow, naming it *Ω*, as

$$\begin{array}{c} \Omega =\sum_{i=1}^{n}\sum_{j=1,j\neq i}^{n}f_{i,j} \end{array}$$ (1)

We assume at most p (0 ≤ *p* ≤ *n* ∗ (*n* − 1)/2) physical links can be removed. We define a binary indicative variable matrix *u*, where *u*_*ij*_ = 1 means “*i* can reach *j* by along link(s)” while *u*_*ij*_ = 0 means “*j* is not reachable from *i*”, i.e., *i* and *j* are disconnected. Since the graph is undirected, *u*_*ij*_ is numerically equal to *u*_*ji*_.

Let *h* be another node in the graph, then according to real world truth, we have the property of *u* as Table 1, where “*” means that the corresponding cell can take a value of either 0 or 1. Explanation of the last line is that, when node *i*, *j* are connected (direct or indirect) and *j*, *h* are connected, then *i*, *h* must be connected. The rest 3 lines means that whether *i* and *h* are connected can’t be deduced out.

**Table 1:** The property of *u*.

*u*_*ij*_	*u*_*jh*_	*u*_*ih*_
0	0	*
0	1	*
1	0	*
1	1	1

This table shows the mathematical condition of u in a graph has to satisfy.

After deleting *p* edges, the network’s performance can be measured using the minimum supported residual flow *Ω*_*p*_.

$$\begin{array}{c} \Omega_{p}=\sum_{i=1}^{n}\sum_{j=1,j\neq i}^{n}u_{i,j}*f_{i,j} \end{array}$$ (2)

The robustness of the network, *Robu*, can be defined as

$$\begin{array}{c} Robu=\Omega_{p}/\Omega \end{array}$$ (3)

*Robu* is between 0 and 1. When residual flow *Ω*_*p*_ is larger, *Robu* is larger and it means that the network is more robust.

Our model is an integer linear programming. The goal is to minimize *Ω*_*p*_ while satisfying two kind of constraints. The first constraint is the number of deletion constraint. One can only delete at most p edges. Written mathematically, the constraint is

$$\begin{array}{c} \sum_{\begin{array}{c} (i,j)\in E\\ i<j \end{array}}\left(1-u_{ij}\right)\leq p \end{array}$$ (4)

The other kind of constraints is that of graphic connectivity. It demands that the variables *u* satisfy the so-called “triangle inequality” as Table 1, so to speak

$$\begin{array}{c} u_{ij}+u_{jh}-u_{ih}\leq 1\;i,j,h\in V \end{array}$$ (5)

In [15], the authors have proven that Eq (5) can be replaced by more efficient constraints as

$$\begin{array}{c} u_{ij}+u_{jh}-u_{ih}\leq 1\;h\in N\left(i\right)\cup N\left(j\right) \end{array}$$ (6)

where *N*(*i*) is the neighbors of node *i* and they have shown the correctness of the substitution.

We call one network (with a determined topology and flow) an “instance” and one calculation of deleting certain edges in this instance a “case”. The robustness of one case of an instance can be formalized as Eq (7) by combining Eqs (2), (4) and (6) together:

$$\begin{array}{c} \begin{array}{ccc} & \text{min} & \sum_{\begin{array}{c} i,j\in V\\ i\neq j \end{array}}u_{ij}f_{ij}\\ & \text{subject}\;\text{to} & u_{ij}+u_{jh}-u_{ih}\leq 1\;h\in N\left(i\right)\cup N\left(j\right)\\ & & u_{ij}=u_{ji}\\ & & \sum_{\begin{array}{c} (i,j)\in E\\ i<j \end{array}}\left(1-u_{ij}\right)\leq p\\ & & u_{ij}=0,1 \end{array} \end{array}$$ (7)

In our experiments, we calculated about 8,000 instances (i.e., networks) and the case number for each is 10, with deletion percentage varying from 5% to 50%. The 8000 instances come from 4 groups; within each group the node number, edge number and flow quantity of instances (networks) are the same— networks are different only in degree distribution, flow distribution and coupling tightness between the two distributions.

In Eq (7), if all *f*_*ij*_ = 1, this problem is called the “critical link set problem (CLP)”. When the p links chosen to be deleted obtain the minimum *Ω*_*p*_, these p links are called the critical links. In the next section, we discuss the hardness of approximation of critical link set problem.

Eq (7) is a global 0-1 integer programming. It can be solved using some mathematical tools/software, but the variable space can be large. We used GUROBI [21] to calculate one group of instances. And for three other groups of instances, we instead used GA for the reasons mentioned before.

We are to build the numerical relations between network robustness and the three factors—the topology, the flow and the coupling. To characterize network’s structure (topology) numerically, we introduce the network structure entropy [22] based on the nodes’ degree sequence. There are also other entropy calculation methods such as [23, 24]. We use the former one because it’s relative simpler in form. For future work, we can experiment the results using the later methods.

Given a graph *G* = (*V*, *E*) and its degree sequence {*d*_*i*_}, 1 ≤ *i* ≤ *n*, the structure entropy of *G* is

$$\begin{array}{c} ET=-I_{i}\ln\left(I_{i}\right); \end{array}$$ (8)

where

$$\begin{array}{c} I_{i}=d_{i}/\sum_{i=1}^{n}d_{i} \end{array}$$ (9)

*I*_*i*_ is called the importance of node *i*. When a graph is very random, the importance of nodes are more likely to be equal, so this graph will be more stable under intentional attack; on the other hand, when some of the nodes in the graph have a large degree, the graph will be more vulnerable, and we would sense that the graph has a small entropy. The smallest entropy graph is a star-like network and the largest entropy graph is a degree-equal graph [22]. Paper [25, 26] also reached similar conclusions. The entropy of any other graph should be within the two extreme cases. Thus, we can normalize the entropy of any graph as Eq (10):

$$\begin{array}{c} \bar{ET}=\frac{ET-ET_{min}}{ET_{max}-ET_{min}}=\frac{2ET-\ln(4(n-1))}{2\ln(n)-\ln(4(n-1))} \end{array}$$ (10)

It is apparent that $0\leq \bar{ET}\leq 1$.

To describe the heterogeneity of the flow, we can define a metric $\bar{EN}$ like $\bar{ET}$, as one unit of interaction flow can be viewed as an arc. Here, we will not bother to write down flow structure entropy $\bar{EN}$ in detail.

A qualifier is needed to quantify the relation between the topology and flow. This qualifier is the coupling coefficient. The terminology can also be named correlation coefficient. If certain node’s degree is relatively large in the graph and its flow degree is also relatively large, then the graph’s topology and flow are positively coupled. We use Spearman correlations [27] to measure the coupling, whose equation is

$$\begin{array}{c} \rho =\frac{\sum {}_{i}(x_{i}-\bar{x})\left(y_{i}-\bar{y}\right)}{\sqrt{\sum {{}_{i}(x_{i}-\bar{x})}^{2}}\sum {}_{i}{\left(y_{i}-\bar{y}\right)}^{2}} \end{array}$$ (11)

where *x*_*i*_ is the rank of node i after sorting according to degree, and $\bar{x}$ is the average rank, which is similar to *y*. *ρ* is within [-1,1].

In a word, we want to discover the relations of *Robu* to $\bar{ET}$, $\bar{EN}$ upon varying *ρ* after deleting 50%, 45%, …, 5% edges. We delete at most 50% of edges because when deleting more edges, the residual flow will be very small, even be zero.

### Hardness

We first attempt to determine the theoretical hardness of the robustness problem before computing. We analyzed a specific case of the robustness problem, the critical link set problem (CLP). The NP completeness of CLP has been proven in [15], so it is impossible to obtain optimal solutions in polynomial time complexity. We now further extend their work to derive the inapproximability ratio of CLP. The inapproximability ratio of a hard-to-tackle problem is the smallest gap between the optimal result and any approximate result obtained by deterministic approximation algorithms. We will show that for CLP, any deterministic algorithms will produce a result larger than 5/3 times of the optimum. Since the robustness problem is harder than CLP, good approximation algorithms do not exist. Hence, for getting near-optimal or optimal result of *Robu*, non-deterministic approaches is the only way. Before deducing our conclusion, certain definitions and lemmas are required.

**Definition 1** [28] *Let* 0 < *α* < *β*. *A minimization problem* *Π* *is said to have an NP-hard gap of* [*α*, *β*] *if there exists an NP-complete problem* Γ *and a polynomial-time many-one reduction* *f* *from* Γ *to* *Π* *with the following properties*:

1. *If* *x* ∈ Γ, *then* *opt*(*f*(*x*)) ≤ *α*, *and*
2. *If* *x* ∉ Γ, *then* *opt*(*f*(*x*)) > *β*.

*where* *opt*() *denotes the optimal objective function value*.

**Lemma 1** *Assume that* *Π* *is an minimization problem with an NP-hard gap* [*α*, *β*], 0 < *α* < *β*. *Then, there is no deterministic polynomial-time* (*β*/*α*)-*approximation algorithms for problem* *Π* *unless P = NP*.

**Proof 1** *Assume that* *f* *is a reduction from an NP-complete problem* Γ *to* *Π* *satisfying properties (i) and (ii) of Definition 1. Suppose, for the sake of contradiction, that there is a polynomial-time* (*β*/*α*)-*approximation A for problem* *Π*. *We may then construct a polynomial-time recognition algorithm for problem* Γ *as follows*:

1. *On inputting instance* *x* *of problem* Γ, *compute the instance* *y* = *f*(*x*) *of problem* *Π*.
2. *Run algorithm A on instance* *y* *to get a* (*β*/*α*)-*approximation S for* *y*.
3. *Return YES if and only if the objective function value of solution S for problem* *Π* *is less than or equal to* *β*.

*It is easy to verify the correctness of the above algorithm: If* *x* ∈ Γ, *then* *opt*(*y*) ≤ *α*, *hence the objective function value of any* (*β*/*α*)-*approximation solution for* *y* *is at most* *α* × *β*/*α* = *β*. *On the other hand, if* *x* ∉ Γ, *then the optimal objective function value of any solution for* *y* *has already been greater than* *β*, *not to mention that the approximation objective value equals the optimal value multiply an approximation ratio that is always greater than 1. Therefore, the approximation value of* *y* *enables us to determine whether* *x* *is in* Γ *whilst* Γ *is an NP-complete problem—this is a contradiction, and we have the proof*.

**Theorem 1** *An approximation algorithm with a ratio less than or equal to 5/3 does not exist for a critical link set problem*.

**Fig 1:**
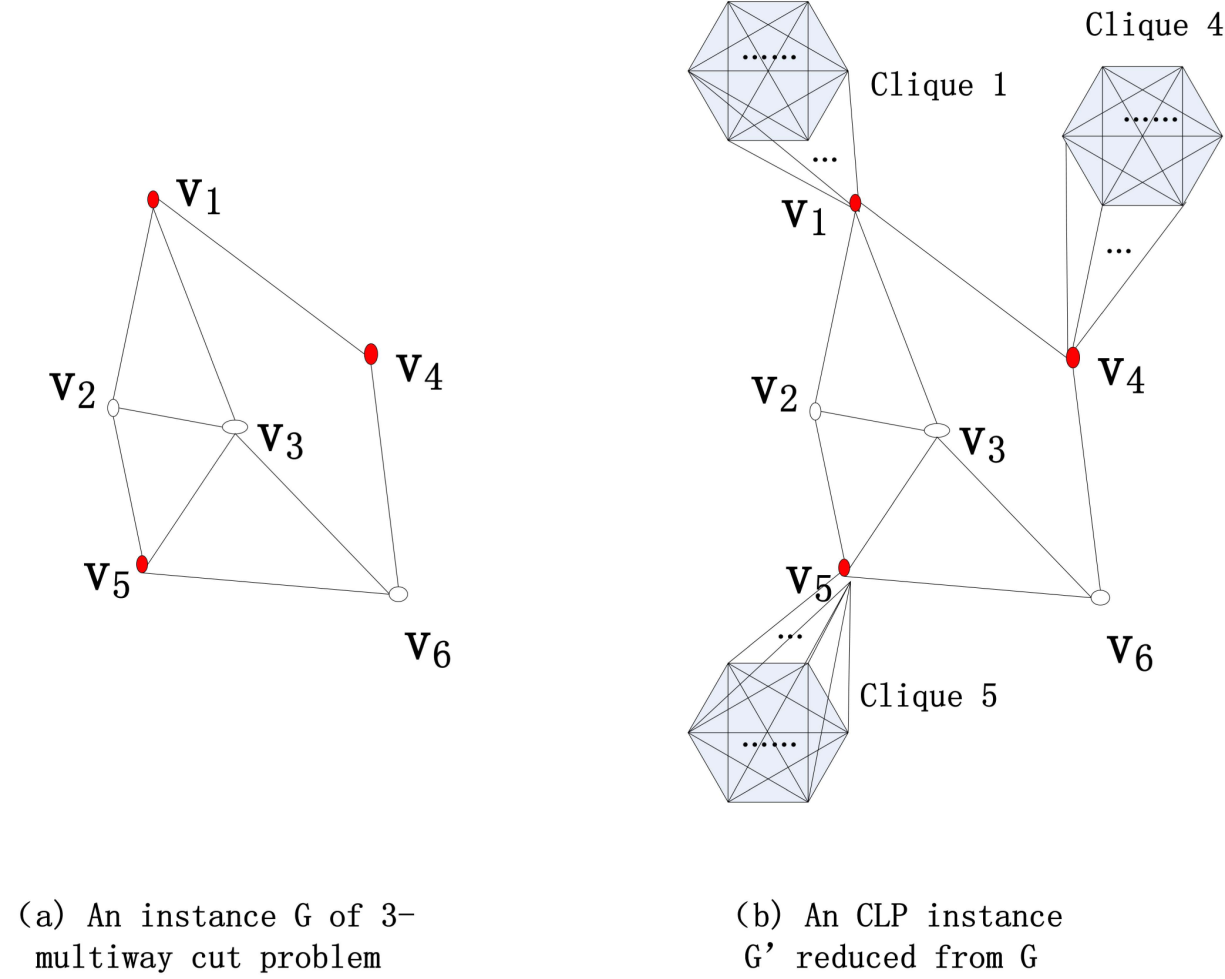
Reduction illustration. The reduction from an instance of 3-multiway cut problem to an instance of CLP.

**Proof 2** *Consider a well-known NP-complete problem, the 3-multiway cut problem. The problem asks if there exists an edge cut set of size k such that the deletion of the set disconnects 3 given nodes (terminals). We can construct a many-one reduction from a 3-multiway cut instance to a CLP instance as* Fig 1. *At each node, we attach a clique of* *n* ^2^ *nodes, whose nodes also connect to the original node, thus forming a clique of* *n* ^2^ + 1 *nodes. Denote the source instance as G and the destination instance as G’. We now try to prove that CLP has a gap* [*α*, *β*], *i.e., if G has a 3-multiway cut of size* *k* *then G’ has a pairwise connectivity at most* *α*, *whereas if G hasn’t a* *k* *size 3-multiway cut, then G’ has at least* *β* *pairwise connectivity. According to the lemma, the inapproximability ratio will be* *β*/*α* *and we need to calculate* *α* *and* *β*.

*We first prove that if G has a 3-multiway cut of size k, then G’ has a pairwise connectivity of at most* $2C_{n^{2}+1}^{2}+C_{n^{2}+n-2}^{2}$ *(“the α”): Let* *S*_*cut*_ *be the set of edges that disconnects the 3 given nodes in G, then* |*S*_*cut*_| = *k*. *S*_*cut*_ *will also disconnect each of the 3 nodes with a clique in G’. Therefore*, *S*_*cut*_ *will partition G’ to at least 3 components, and no component will contain more than 1 clique. A previous article* [29] *has proven that fewer components results in greater pairwise connectivity. Therefore, when* *S*_*cut*_ *partitions G’ to 3 parts, the total pairwise connectivity will be the greatest. Suppose* *S*_*cut*_ *cuts G’ to 3 parts with a size of* (*n* ^2^ + 1) + *x*_*i*_, i = 1, 2, 3, *x*_*i*_ ≥ 0, *x*_1_ + *x*_2_ + *x*_3_ = *n* − 3, *and this is the best partition strategy. Under this circumstance, the maximization of the total pairwise connectivity is equivalent to the minimization of the pairwise connectivity loss, where the loss is simply caused by the disconnection of nodes in different components. So*

$$\begin{array}{ccc} loss & = & \left[\left(n^{2}+1\right)+x_{1}\right]*\left[\left(n^{2}+1\right)+x_{2}\right]+\left[\left(n^{2}+1\right)+x_{1}\right]*\left[\left(n^{2}+1\right)+x_{3}\right]\\ & & +\;\left[\left(n^{2}+1\right)+x_{2}\right]*\left[\left(n^{2}+1\right)+x_{3}\right]\\ & = & CONSTANT+x_{1}*x_{2}+x_{1}*x_{3}+x_{2}*x_{3} \end{array}$$

*When two of the 3 x’s take value 0, the loss will be the smallest, which is equal CONSTANT, and thus the pairwise connectivity after partition is the greatest. That is to say, G’ is parted to* *n* ^2^ + 1, *n* ^2^ + 1, (*n* ^2^ + 1) + (*n* − 3), *and the pairwise connectivity is* $2C_{n^{2}+1}^{2}+C_{n^{2}+n-2}^{2}$.

*Conversely, we can show that if G does not have a k size 3-multiway cut, then G’ has at least* $C_{2n^{2}+2}^{2}+C_{n^{2}+n-2}^{2}$ *(“the β”): if G does not have a 3-multiway cut, then G might have been parted to 1 or 2 components, and obviously being parted to 2 components has less pairwise connectivity. Similar to considering the loss that was previously defined, we know that the optimum occurs when the size of the 2 components are* 2 ∗ (*n* ^2^ + 1), *n* ^2^ + *n* − 2. *Therefore the inapproximability ratio is*:

$$\begin{array}{ccc} \rho^{\prime } & = & \beta /\alpha =\frac{C_{2n^{2}+2}^{2}+C_{n^{2}+n-2}^{2}}{2C_{n^{2}+1}^{2}+C_{n^{2}+n-2}^{2}}\\ & = & \frac{\frac{\left(2n^{2}+2\right)\left(2n^{2}+1\right)}{2}+\frac{\left(n^{2}+n-2\right)\left(n^{2}+n-3\right)}{2}}{2*\frac{\left(n^{2}+1\right)n^{2}}{2}+\frac{\left(n^{2}+n-2\right)\left(n^{2}+n-3\right)}{2}}\\ & = & \frac{5n^{4}+2n^{3}+2n^{2}-5n+8}{3n^{4}+2n^{3}-2n^{2}-5n+6}=\frac{5}{3}\left(Omitting\;the\;lower\;order\right) \end{array}$$ (12)

This means that there is likely to be no polynomial-time **deterministic** $\frac{5}{3}$-approximation algorithm for CLP. It also means the robustness problem is difficult to approximate too. However, the need for knowing the robustness of a network is demanding, so we have to develop estimation approaches for robustness. This desire drives us to empirically study the relations among robustness and factors using genetic algorithms, and then fortunately find the near linearity relations.

### Data and Experiments

Because there were no analytic conclusions for the robustness and related factors, we decided to study their discrete relation in a empirical way. In this section, we described how we generated the required instances and how we performed computation on them. The generation steps are as follows:

1) Given a node and edge scale, generate networks with different structure entropies.

2) Given a flow scale, on the graphs of step 1), to generate flow matrix with different entropies.

3) Relabel the indices of the nodes in the second step so that the topology distribution and flow distribution produces different correlation coefficient.

To generate an adjacency matrix with specified $\bar{ET}$, rewiring link techniques are needed. According to [22], if the entropy is greater than desired, we rewire edges from a centralized node to small degree node; otherwise perform an inversion until the err is tolerable.

We ultimately generated four groups of instances (See S1 Appendix). The group names are 50-200-1000, 50-200-10000, 50-600-10000, 87-200-4000. The first number is the node size, the second the edge size and the last is the flow size. In each group, we generated networks with structural entropy and flow entropy from 0.1 to 1.0 with step length 0.1. The Spearman coefficients are from -1.0 to 1.0, interval 0.1, so there are 10 ∗ 10 ∗ 20 = 2000 networks (instances) in each group.

After the generation comes the computation.

4) On each generated instance, to calculate 10 cases—corresponding to deleting 50%, 45%, …, 5% edges (floored if not integral). For each case, use exact algorithms or a high performance evolutionary algorithm.

5) Analyze the relations statically after gathering the result data together. For example, in group 50-200-1000, we will analyze what the relations among *Robu*, “graph entropy” and “flow entropy”, if we delete 20% of edges while spearman correlation is strongly negative.

For the computation, we used GUROBI for the group 50-200-1000. GUROBI is thought to be the most efficient integer programming software, but it still costs too much time for our problem. This software uses exhaustive search methods such as cutting-plane techniques [30] for integer linear programming and it provides interfaces for programming languages to call. We also designed an ordinary genetic algorithm to compute this group. After comparison, we found that GA performs well too, thus we adopted GA for the other groups of the instances because GUROBI is too time consuming.

We have not included a detailed description of the generation and computation algorithms here, but we can provide it upon request to readers with interests.

## Results and Discussion

**Fig 2:**
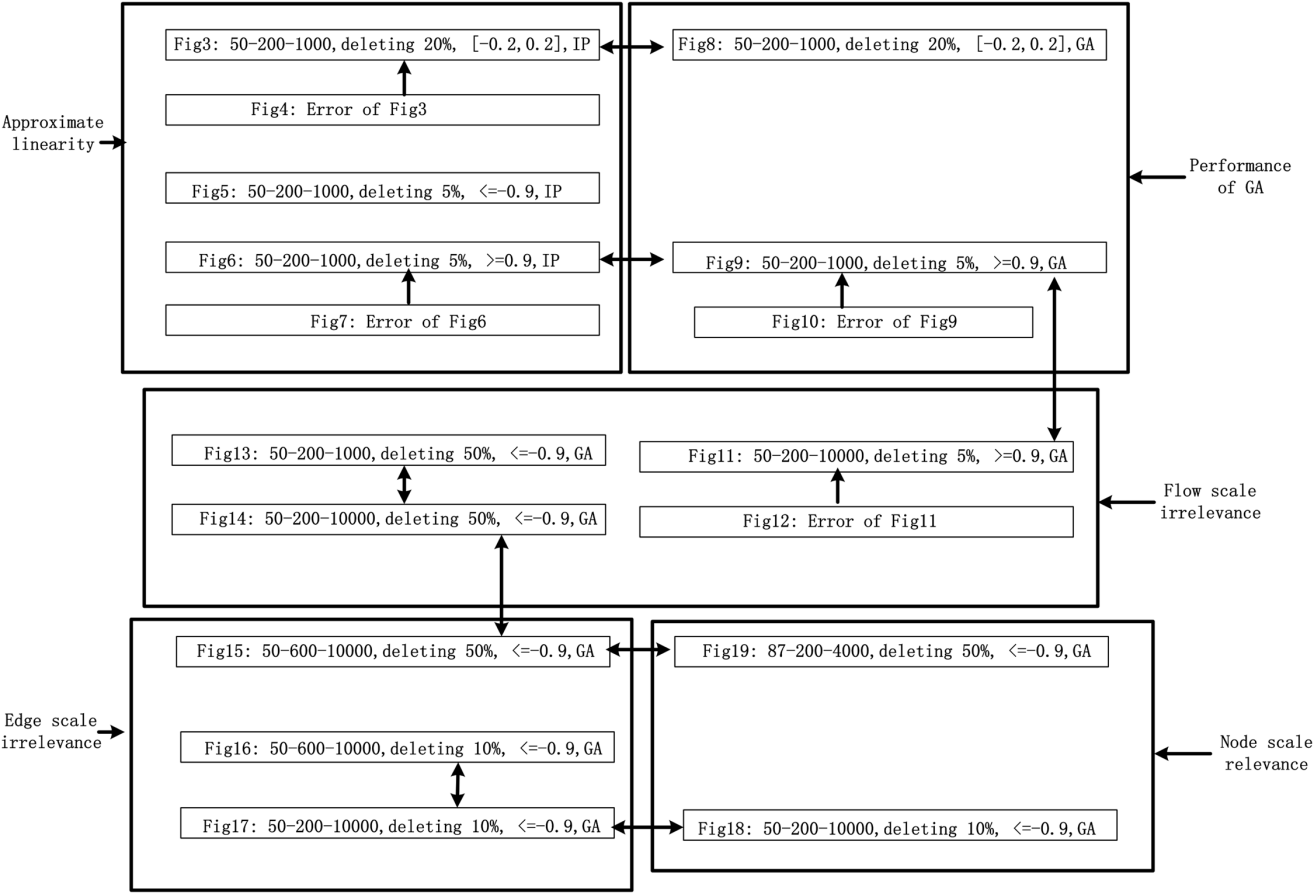
Relations of all figures. The relations of all figures: comparisons and complementary.

**Fig 3:**
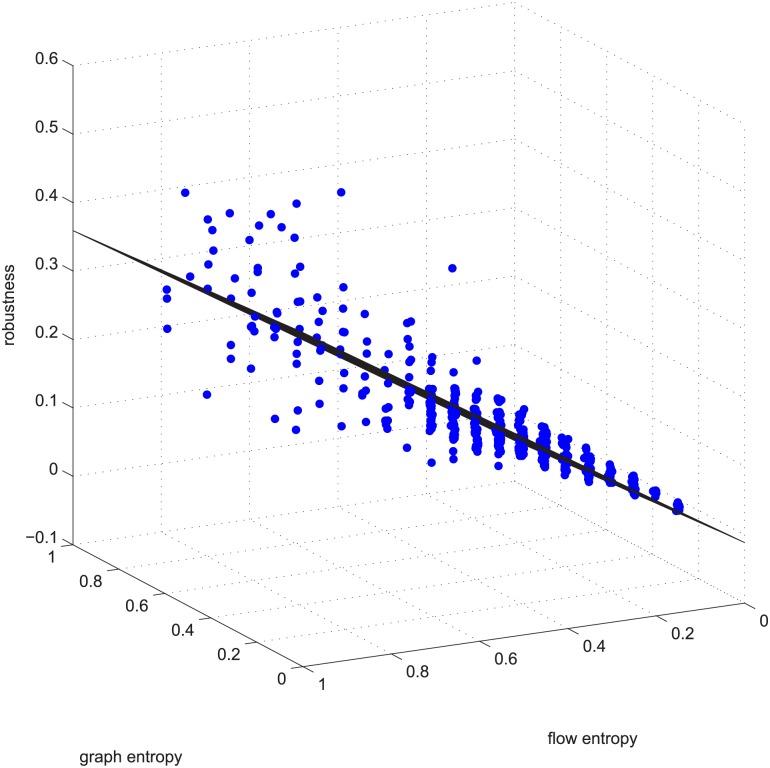
Linear relation example 1. Example 1 is deleting using GUROBI 20% of the edges in [−0.2, 0.2] coupled networks in group 50-200-1000.

The logic of this part is more or less mentioned before: at first, we discover the linearity of relations, so we showed 3 examples for text length. Then, we turned to GA and found the regression coefficients were near to those of integer programming (IP), that’s to say GA is capable for usage. And then, we tested the impacts of flow scales, edge scale and node scales. At last, we utilized the historical coefficients to real world network robustness computation and got favorable results. The picture of Fig 2 would explain more. “Fig 3: 50-200-1000, deleting 20%, [-0.2, 0.2], IP” means Fig 3 is for the 50-200-1000 dataset, deleting percentage 20%, results for networks whose Spearman coefficients are within [-0.2, 0.2], using integer programming. The bidirectional arrows links two samples that to compare, while the unidirectional arrows means that the origin is a complementary to the terminate. In each black box, there are at least two examples to support our conjecture.

### Approximate Linearity for Exact Results

**Fig 4:**
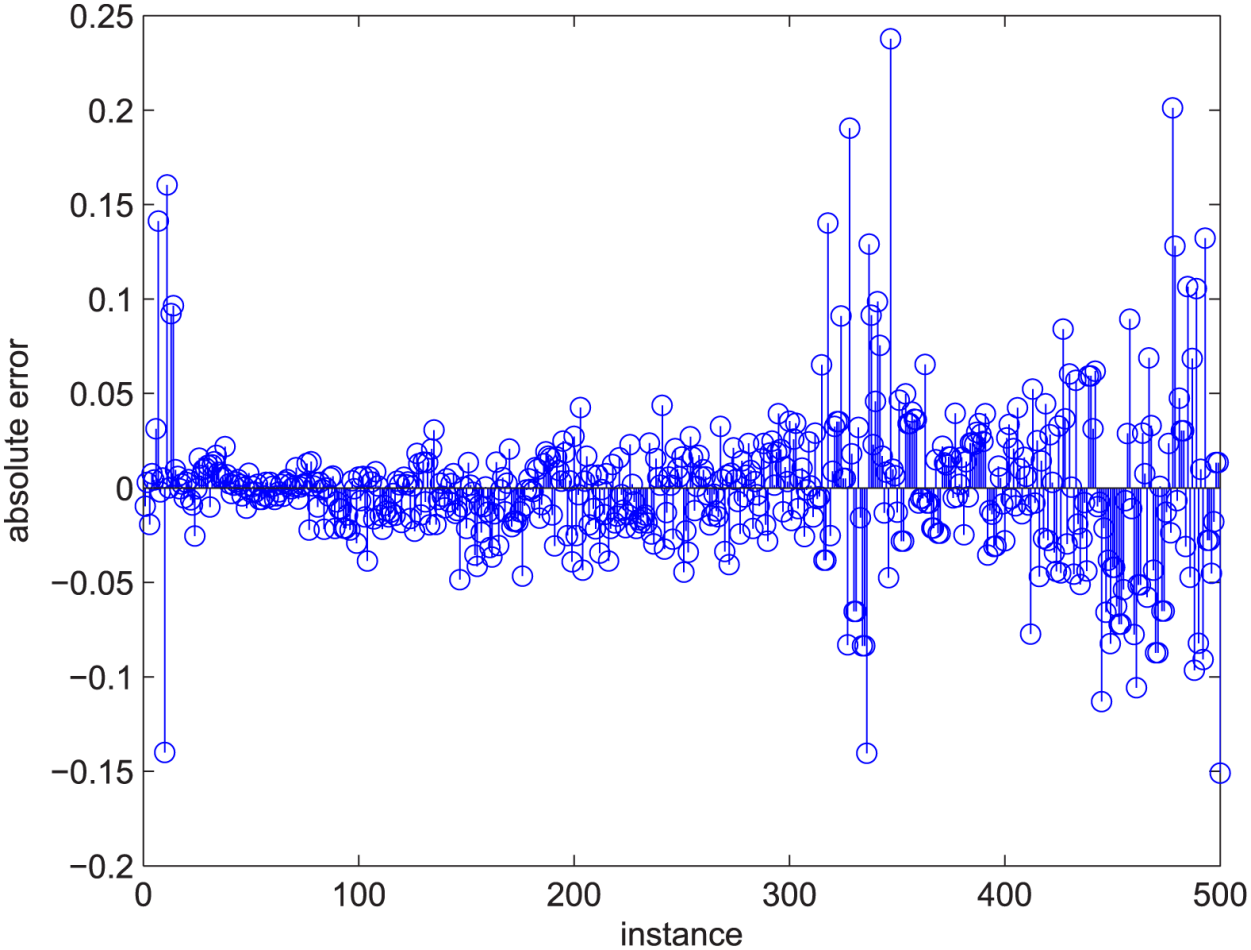
Fig 3’s absolute error. The stem graph for the absolute error between each data point and the regression plane in Fig 3.

Figs 3 and 4 are for the 50-200-1000 group when deleting 20% of the edges using the exact algorithm, and the absolute value of the Spearman correlation is smaller than 0.2, which means the degree distribution and flow distribution are “loosely coupled”. There are 500 data in this category. The Z-axis is the robustness value, and the X-axis and Y-axis are the entropies. The data form a linear regression because the data are now nearly planar. The plane is now observed like a line because of our visual angle. Most of the absolute error is within [-0.05, 0.05].

**Fig 5:**
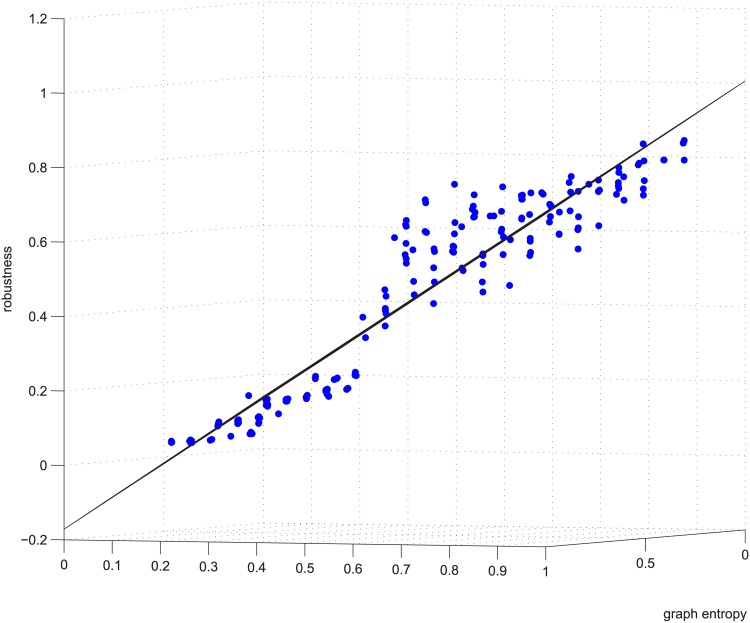
Linear relation example 2. Example 2 is deleting **using GUROBI** 5% of the edges in the ≤ −0.9 coupled networks in group 50-200-1000.

Fig 5 is another example of linearity. Fig 5 is the data for deleting 5% of the edges in group 50-200-1000, with a Spearman value smaller than -0.9 (strongly negatively coupled). It’s regression vector is [0.140 -0.312 0.867], i.e., $robu=0.140-0.312*\bar{ET}+0.867*\bar{EN}$.

**Fig 6:**
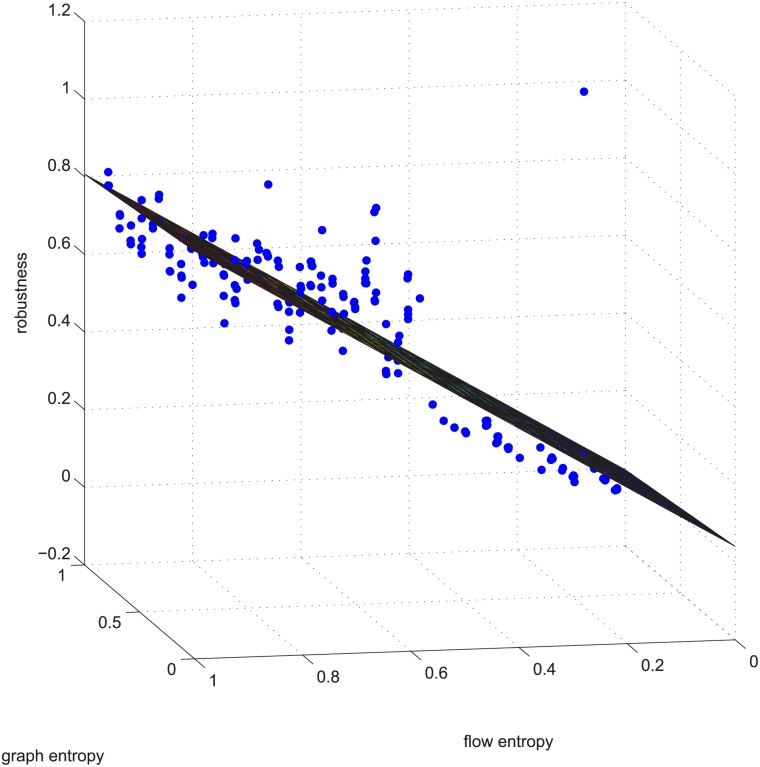
Regression example 2 of GUROBI-computed. This example is deleting using GUROBI 5% of the edges in ≥0.9 coupled networks in group 50-200-1000.

**Fig 7:**
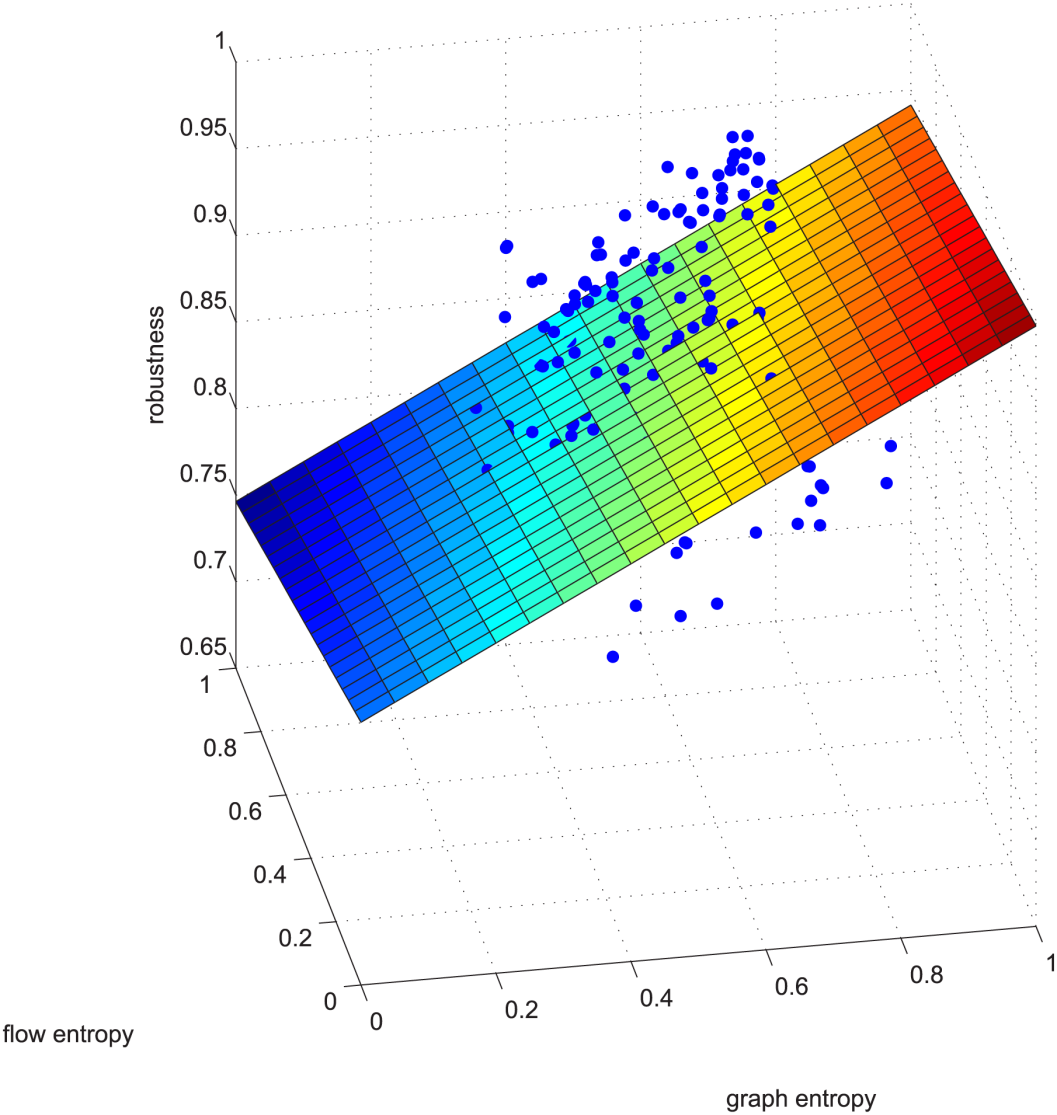
Relative error for regression example 2 of GUROBI-computed. Fig 6’s relative error.

Figs 6 and 7 are a third supporting example. For text length, we are not to show more. Figs 6 and 7 will be used later.

### Performance of GA

**Fig 8:**
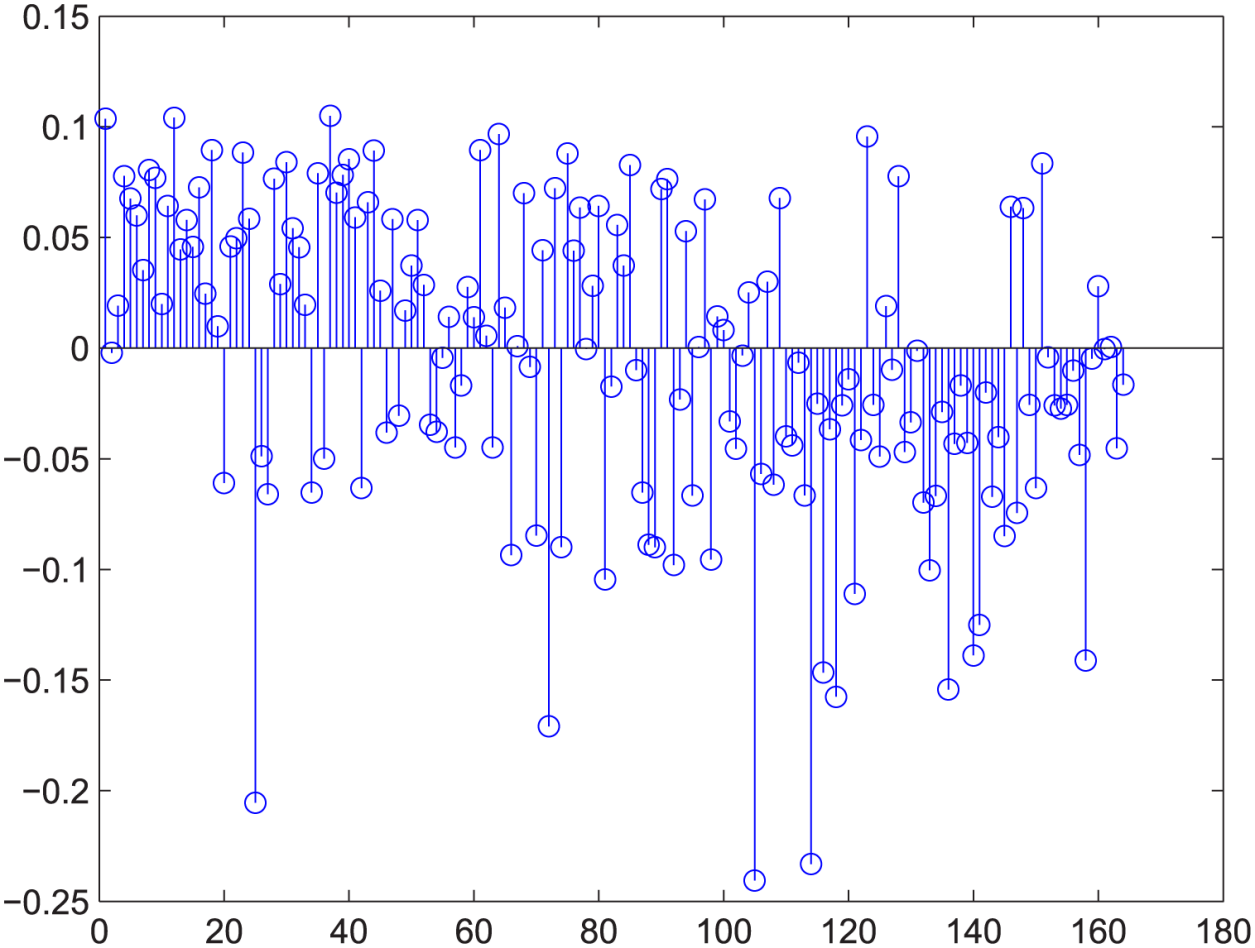
Regression example 1 of GA-computed. This example is deleting **using GA** 5% of the edges in ≤ −0.9 coupled networks in group 50-200-1000.

As mentioned previously, the exact algorithm is very time-consuming, so we tried using a GA. From Fig 8 we can see that the GA data also forms a plane, a plane with similar coefficients. The regression vector is [0.120 -0.144 0.810], which is near to Fig 3’s regression vector([0.140 -0.312 0.867]).

**Fig 9:**
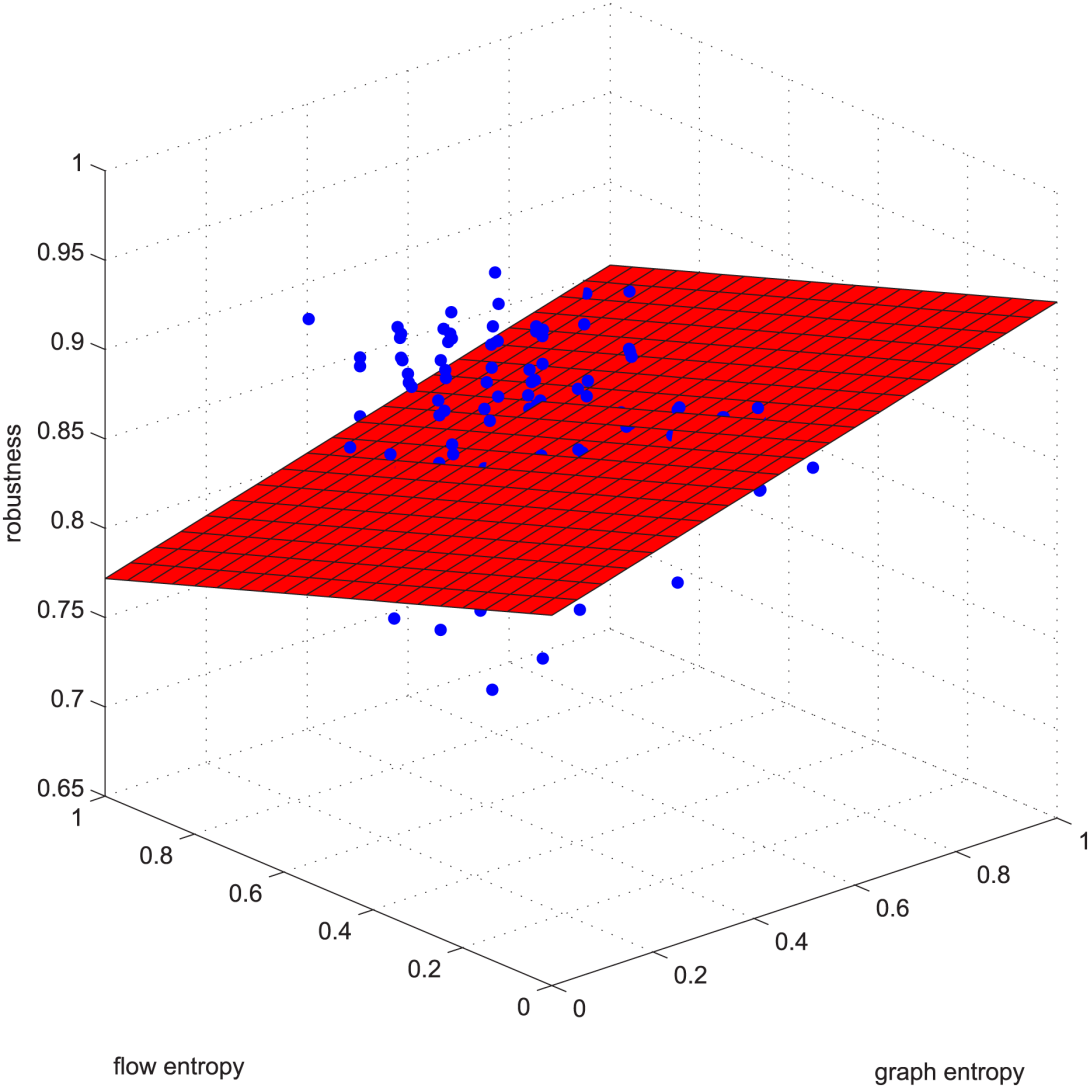
Regression example 2 of GA-computed. This example is deleting using GA 5% of the edges in ≥0.9 coupled networks in group 50-200-1000.

**Fig 10:**
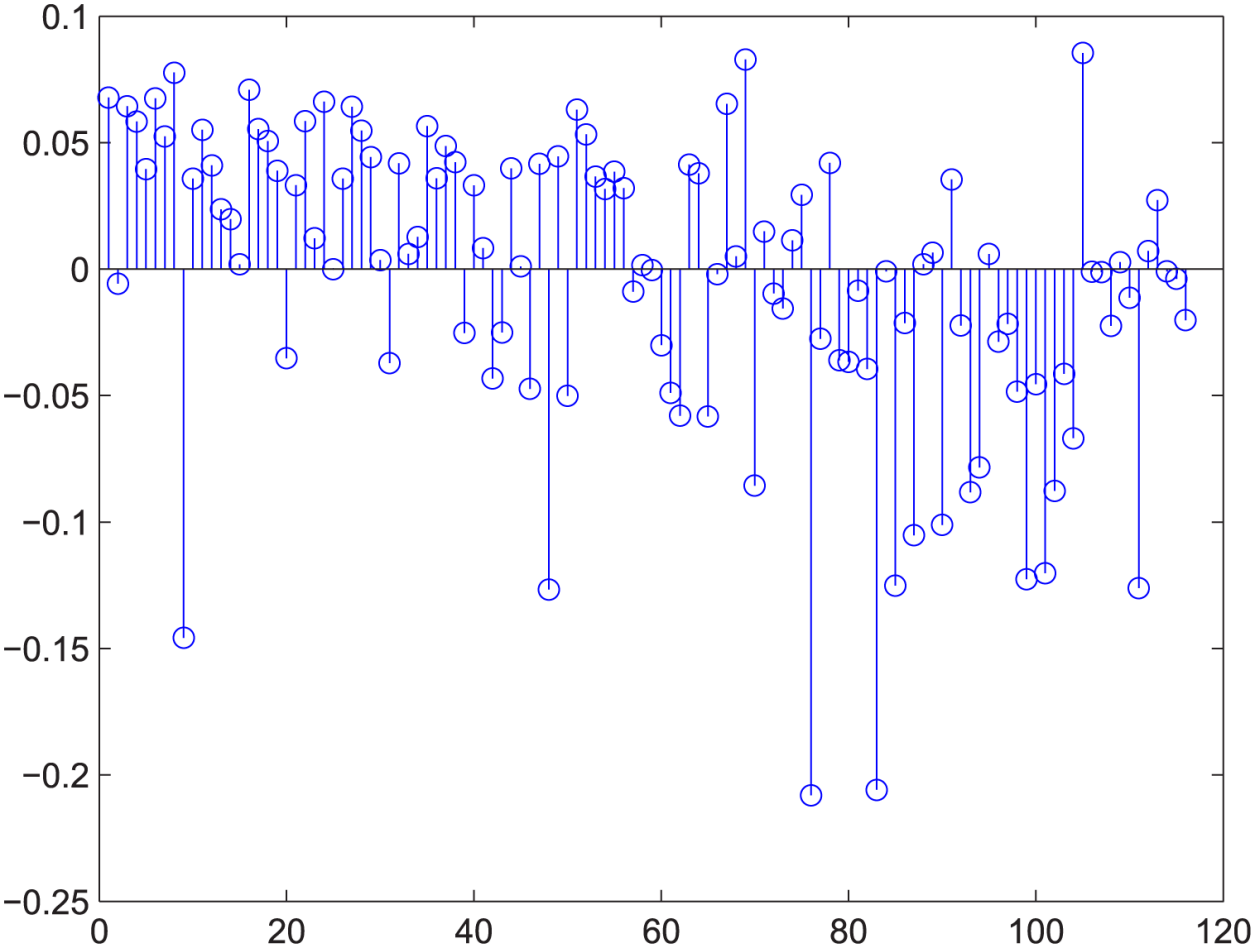
Relative error for regression example 2 of GA-computed. Fig 9’s relative error.

There are additional supports for resorting to GA, such as Figs 9 and 10. The two figures correspond to Figs 6 and 7. These four figures demonstrate deleting 5% of the edges in strongly positively coupled networks using GUROBI and GA. The data fit a plane very well and the regression vector for GUROBI is [0.802 0.0817 -0.05], whereas for GA, the vector is [0.857 0.1950 -0.04]. The relative error is almost within ±10%. The two vectors are similar, just as in the former example. Based on these facts, we believe that our genetic algorithm’s results are close to the optimal. So we adopted GA for the rest of the computations for the massive computations of our experiments.

### Irrelevance of Flow Scale

**Fig 11:**
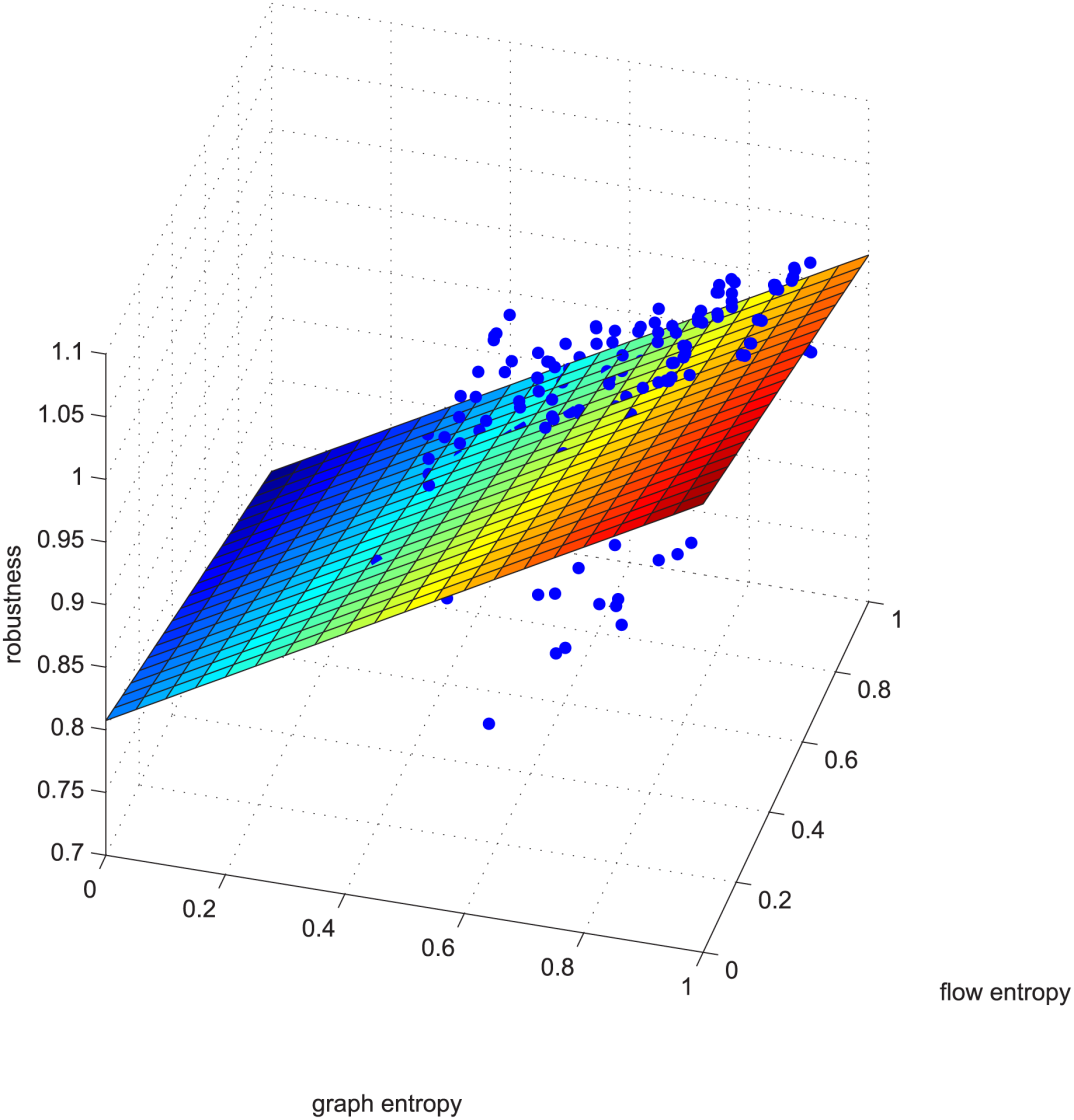
Flow irrelevance example 1. This example is deleting using GA 5% of the edges in ≥0.9 coupled networks in group 50-200-10000.

**Fig 12:**
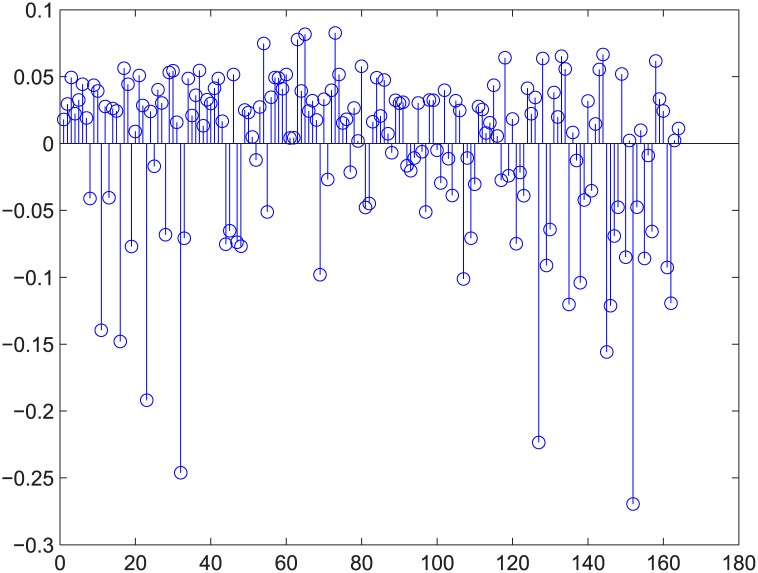
Relative error for the example. Fig 11’s relative error.

We compared the results of group 50-200-10000 to 50-200-1000 with other parameters fixed. The regression coefficients for this example (Figs 11 and 12) are [0.808 0.250 -0.081], which are similar to those of Fig 9 ([0.857 0.1950 -0.04])— the flow scale expanded 10 times but the coefficients are near, because we normalized the robustness to within [0,1].

**Fig 13:**
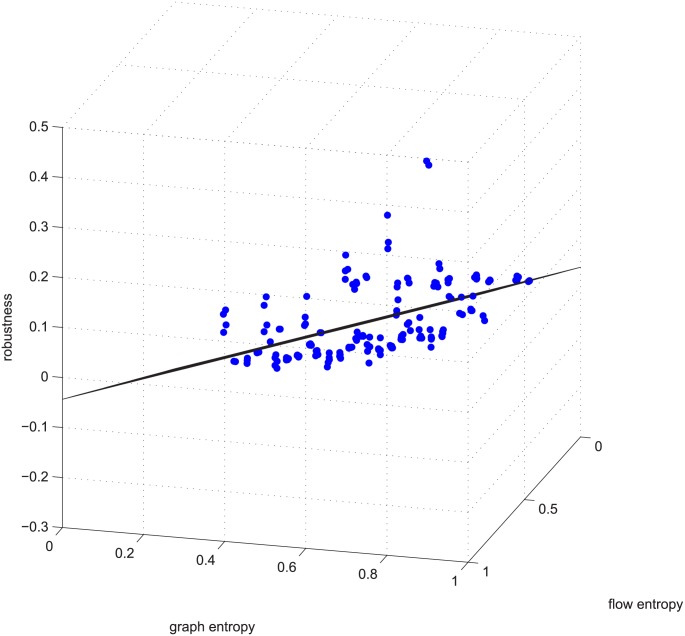
Flow irrelevance example 2. This example is deleting using GA 50% edges in ≤ −0.9 coupled networks in group 50-200-1000.

**Fig 14:**
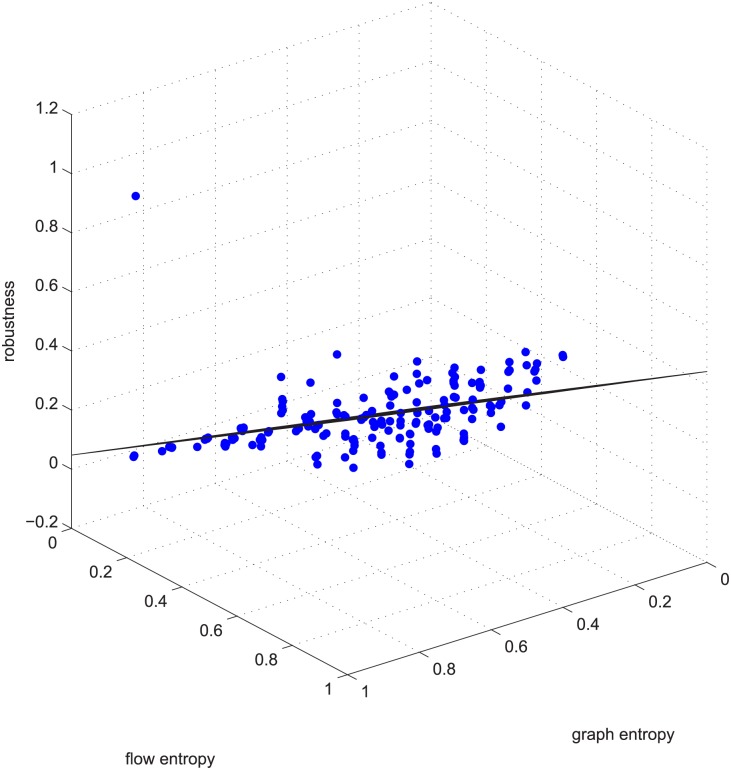
Flow irrelevance example 2. This example is deleting using GA 50% edges in ≤ −0.9 coupled networks in group 50-200-10000.

This is not a singular phenomenon. Figs 13 and 14 display two samples that differ only in the flow scale. Regression vector for the two are [-0.215 0.239 0.088] and [-0.233 0.272 0.190]. We can see that the difference is slight too. So we conclude the flow magnitude does not affect our model’s robustness of networks.

### Irrelevance of Edge Scale

**Fig 15:**
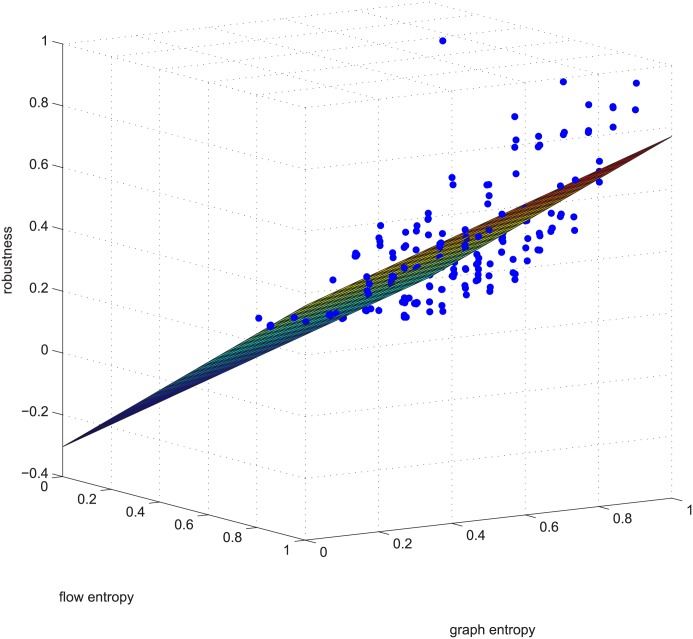
Edge irrelevance example 1. This example is deleting using GA 50% edges in ≤ −0.9 coupled networks in group **50-600-10000**.

Because we delete edges by percentage, we will want to know whether the robustness relates to the edge size. The experiments find that, no matter in dense or sparse graph, once we delete the same percentage of the edges, the resulting robustness is alike. There are many supporting data. Fig 15 give an illustration, its vector is [-0.290 0.356 0.205], just similar to that of Fig 15 ([[-0.233 0.272 0.190]).

**Fig 16:**
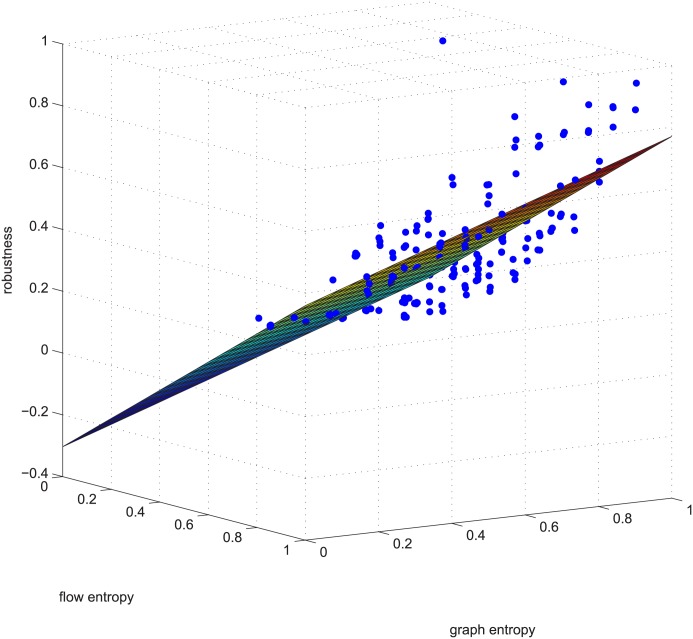
Edge irrelevance example 2. This example is deleting using GA 10% of the edges in ≤ −0.9 coupled networks in group 50-600-10000.

**Fig 17:**
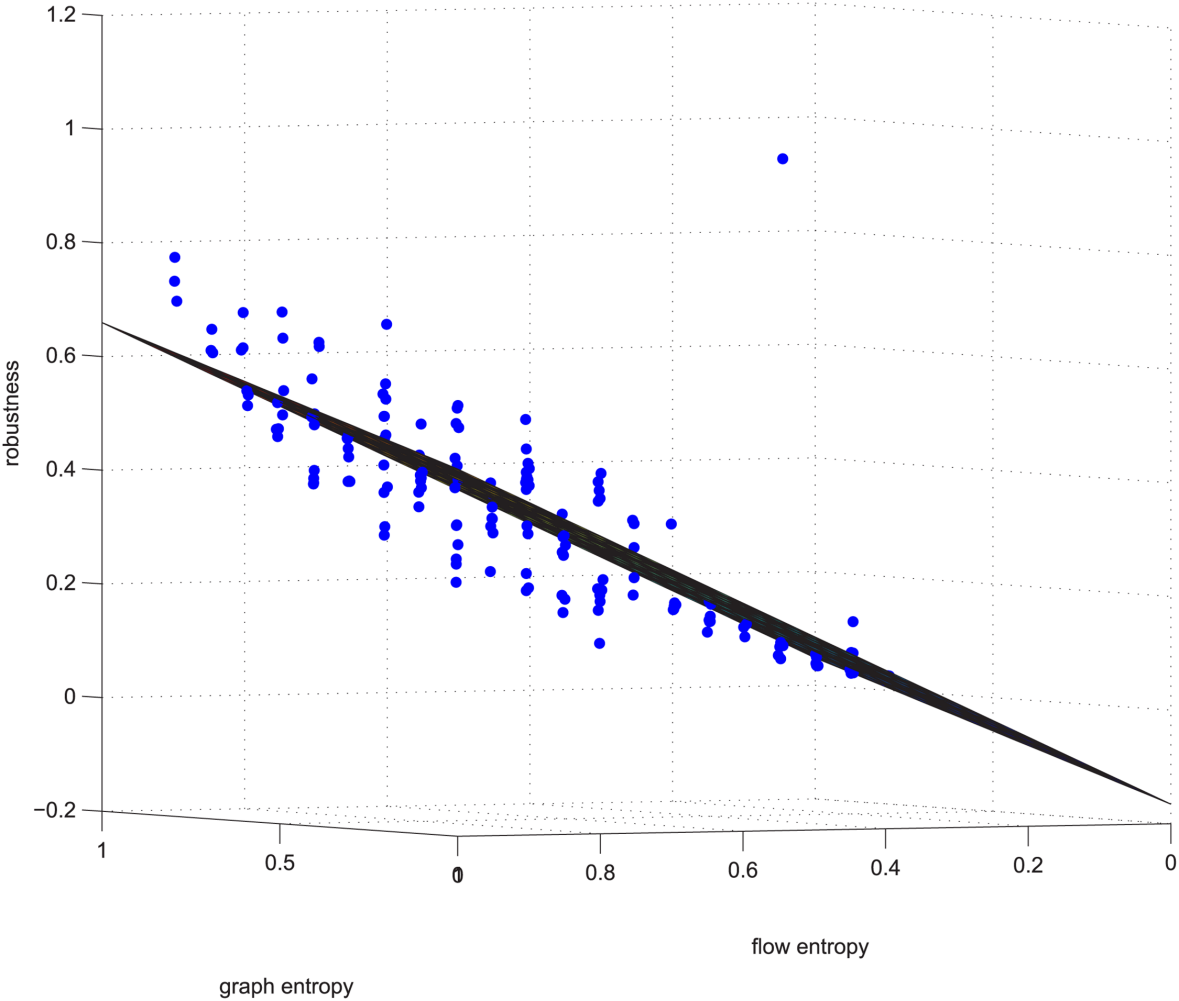
Edge irrelevance example 2. This example is deleting using GA 10% of the edges in ≤ −0.9 coupled networks in group 50-200-10000.

Figs 16 and 17 provide another illustration. Their vectors are [-0.366 0.214 0.6113] and [-0.303 0.412 0.662].

### Relevance of Node Scale

**Fig 18:**
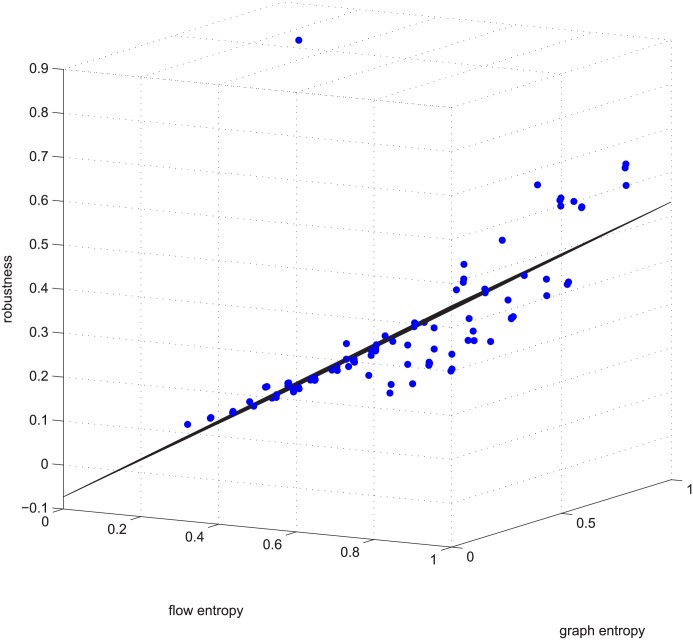
Node relevance example 1. This example is deleting using GA 50% of the edges in ≤ −0.9 coupled networks in group 87-200-4000.

**Fig 19:**
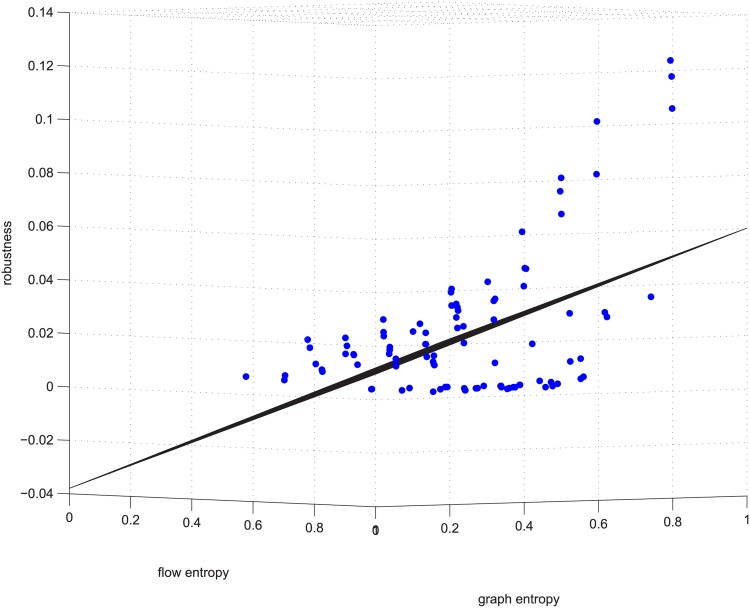
Node relevance example 2. This example is deleting using GA 10% of the edges in ≤ −0.9 coupled networks in group 87-200-4000.

Finally, we want to know whether the node size matters. To our findings, node size truly matters. For example Fig 18’s vector is [-0.073 0.083 0.522], which is quite different from that of Fig 17. And the vector of Fig 19 is [-0.038 0.050 0.048], which is also quite different from Fig 15.

### Real-world Application

Because the flow size and the edge size do not matter in the model, we can store each coefficients vector for node size 50, considering certain Spearman relation together with a certain deletion percentage. That is, we store a dictionary *D*: *R* × *R* → *R* × *R* × *R*. The left two real numbers are (Spearman correlation, deletion percentage), and right three numbers are coefficients vector (*v*0*v*1*v*2), where *v*0 is constant coefficient, *v*1 is coefficient for topology entropy, *v*2 for flow entropy. On receiving a deletion case with *same node size*, we can estimate its robustness after certain deletion using the stored vectors, step by step:

1. calculate the topology entropy of the network *EN*.
2. calculate the flow entropy of the network *ET*.
3. calculate the Spearman correlation of the network.
4. Round the number of edges for deletion to nearby percentage in our dictionary.
5. Find the coefficients vector (*v*0*v*1*v*2) mapped by duple (correlation, deletion percentage), together with the parameters calculated in 1) 2), thus obtain the rough estimation by multiplying vector by [1*ENET*], i.e. *Robu* = (*v*0, *v*1, *v*2) ⋅ [1*ENET*]^*T*^.

To validate this conjunction, we carried computation on a real-world dataset and the results proved the conjunction to be effective and efficient. We found a well-known dataset with 50 nodes, 100 edges, 2450 unit’s flow. The dataset which is from a Operational Research data library in http://people.brunel.ac.uk/mastjjb/jeb/orlib/files/, is named “steinb4.txt”. We chose this file because its node size is the same with our empirical studies before and that the robustness of a steiner graph is also a want-to-know by Steiner problem dealers. We set the edge weight to be 1 and thus this graph has a normalized structural entropy ET = 0.906. We let each pair of node has a flow of 1 unit and thus the normalized flow entropy EN = 1.000, thus the spearman correlation is 0.

The historical regression vector (coefficients) of deleting 5% to 50% edges is as follow, when the coupling correlation is very small (absolute value of Spearman Correlation smaller than 0.2, so we can use the following vector because the real-world network’s correlation is 0):

5%:[0.170 0.002 0.723]

10%:[0.014 -0.023 0.648]

15%:[-0.011 -0.052 0.550]

20%:[-0.011 -0.029 0.400]

25%:[-0.011 -0.011 0.285]

30%:[-0.011 -0.004 0.208]

35%:[-0.011 0.003 0.147]

40%:[-0.009 0.002 0.105]

45%:[-0.007 0.002 0.071]

50%:[-0.006 -0.000 0.050]

We can estimate a network’s robustness by calculate the inner product of the vector and [1*ETEN*]^*T*^. Table 2 shows our results. Line 1 is the number of deleted edges. Line 2 is the nearest deletion percentage. Line 3 is the exact residual flows by Gurobi [21] and Line 4 is the exact robustness. Line 5 is the estimated robustness using our approximation linearity equation. And the last line is the absolute error between exact robustness and estimated robustness. We can see that most of the entries’ error is less than 0.02 and some of the entries is even equal. So the estimation can be thought to be effective.

**Table 2:** Real-world applications.

deletion	3	10	20	30	40	52
percent	5%	10%	20%	30%	40%	50%
exact residual flow	1081	1524	890	476	302	146
exact robu	0.882	0.622	0.363	0.194	0.123	0.059
regression value	0.894	0.641	0.363	0.193	0.116	0.044
error	0.012	0.019	0.000	0.001	-0.007	-0.015

## Conclusion

In this paper, we analyzed the hardness of a special case of the robustness model and empirically studied the relations between robustness and topology, flow and their coupling level. This work considers more factors that contribute to robustness variation in complex networks than the previous literature have considered. The findings are novel and can be used in situations where slight error is tolerable. By applying the historical data to a very different real world networks, the effectiveness of estimation approach is verified. In the future, we would like to explore other methods of characterizing the degree and flow distribution and to compare which methods produces more preciser estimation. Moreover, we want to establish a more realistic robustness model in future.

## Supporting Information

S1 Appendix The dataset and results. This appendix contains the dataset we generated, and on them we carried our experiments. We collected the results into the .xml file and analysed the results.
(ZIP)
